# A Systematic Molecular Pathology Study of a Laboratory Confirmed H5N1 Human Case

**DOI:** 10.1371/journal.pone.0013315

**Published:** 2010-10-12

**Authors:** Rongbao Gao, Libo Dong, Jie Dong, Leying Wen, Ye Zhang, Hongjie Yu, Zijian Feng, Minmei Chen, Yi Tan, Zhaojun Mo, Haiyan Liu, Yunyan Fan, Kunxiong Li, Chris Ka-Fai Li, Dexin Li, Weizhong Yang, Yuelong Shu

**Affiliations:** 1 Department of Influenza, Chinese National Influenza Center, State Key Laboratory for Molecular Virology and Genetic Engineering, National Institute for Viral Disease Control and Prevention, Chinese Center for Disease Control and Prevention (China CDC), Beijing, China; 2 Office for Disease Control and Emergency Response, Chinese Center for Disease Control and Prevention (China CDC), Beijing, China; 3 Department of Infectious Diseases, Center for Disease Control and Prevention of Guangxi Zhuang Autonomous Region, Nanning, China; 4 Department of Infectious Diseases, Nanning City Center for Disease Control and Prevention, Nanning, China; 5 First Affiliated Hospital of Guangxi Medical University, Nanning, China; 6 MRC Human Immunology Unit, The Weatherall Institute of Molecular Medicine, John Radcliffe Hospital, University of Oxford, Oxford, United Kingdom; University of Cambridge, United Kingdom

## Abstract

Autopsy studies have shown that human highly pathogenic avian influenza virus (H5N1) can infect multiple human organs other than just the lungs, and that possible causes of organ damage are either viral replication and/or dysregulation of cytokines and chemokines. Uncertainty still exists, partly because of the limited number of cases analysed. In this study, a full autopsy including 5 organ systems was conducted on a confirmed H5N1 human fatal case (male, 42 years old) within 18 hours of death. In addition to the respiratory system (lungs, bronchus and trachea), virus was isolated from cerebral cortex, cerebral medullary substance, cerebellum, brain stem, hippocampus ileum, colon, rectum, ureter, aortopulmonary vessel and lymph-node. Real time RT-PCR evidence showed that matrix and hemagglutinin genes were positive in liver and spleen in addition to positive tissues with virus isolation. Immunohistochemistry and in-situ hybridization stains showed accordant evidence of viral infection with real time RT-PCR except bronchus. Quantitative RT-PCR suggested that a high viral load was associated with increased host responses, though the viral load was significantly different in various organs. Cells of the immunologic system could also be a target for virus infection. Overall, the pathogenesis of HPAI H5N1 virus was associated both with virus replication and with immunopathologic lesions. In addition, immune cells cannot be excluded from playing a role in dissemination of the virus in vivo.

## Introduction

Influenza pandemic are characterized by the worldwide spread of novel influenza strains for which most of the population lacks substantial immunity [1], [2]. Pandemic viruses typically cause heightened morbidity and mortality [1]. The continued circulation of highly pathogenic avian influenza (HPAI) viruses H5N1 has resulted in occasional coincident infections among humans. Since late 2003, when widespread H5N1 virus poultry outbreaks were reported in multiple countries in Asia, there have been 467 laboratory confirmed human cases in ten countries reported to the World Health Organization as of December 2009 with a mortality rate of about 60% [3], [4]. Global public health concerns surrounding H5N1 viruses include not only individual transmission events between infected poultry and individual humans, but also their pandemic potential, should these viruses acquire genetic changes that result in sustained human-to-human transmission. To date, several case clusters of H5N1 infections have been reported [5] and limited epidemiologic information has suggested person-to-person transmission of H5N1 in a few instances, usually involving family members. Of additional concern to both human and animal health, is the extensive geographic spread of HPAI H5N1 viruses in recent years and their isolation from multiple species of wild birds and mammals [6]–[9]. Despite the recent emergence of the 2009 H1N1 pandemic [10], the pandemic threat from HPAI H5N1 viruses has not diminished [11].

Human H5N1 disease is clinically and pathologically distinct from that caused by seasonal human influenza A H3N2 or H1N1 viruses [12]. The majority of confirmed human HPAI H5N1 virus infections have been characterized by a severe clinical syndrome including a rapid progression of lower respiratory tract disease, often requiring mechanical ventilation within days of admission to a hospital [13]–[18]. In addition to pulmonary complications, other clinical manifestations of H5N1 virus infections may include severe lymphopenia, gastrointestinal symptoms, and liver and renal dysfunction [14], [16], [17], [19], [20]. Reactive hemophagocytosis in multiple organs, and occasional detection of viral antigen or viral RNA in extrapulmonary organs suggest a broader tissue distribution of H5N1 viruses compared with seasonal viruses in fatal human cases [21], [22].

Patients with severe H5N1 disease have unusually higher serum concentrations of proinflammatory cytokines and chemokines. Levels of plasma macrophage attractant chemokines CXCL10 (IP-10), CXCL9 (MIG), and CCL-2 (monocyte chemoattractant protein 1, MCP-1) and of neutrophil attractant interleukin-8 (IL-8) were substantially higher in patients with H5N1 disease compared with those experiencing seasonal influenza virus and were significantly higher in H5N1 patients who died compared with those who recovered [23], [24]. The elevation of plasma cytokine levels was positively correlated with pharyngeal viral load [23] and may simply reflect more extensive viral replication, and consequently, direct viral pathology rather than being causative of the pathology observed in H5N1-infected patients. Compared with human H1N1 and H3N2 influenza viruses, infection of human primary macrophage cultures in vitro with H5N1 viruses also lead to the hyper-induction of proinflammatory cytokines [25].

Most studies on H5N1 pathology describe pulmonary features of human disease. Although H5N1 virus infection of humans is primarily one of the lower respiratory tract, more recent reports suggested that influenza A H5N1 may in rare, severe cases, disseminate beyond the lungs and infect brain [26], [27], intestines [20], [27] and lymphoid tissues [27], and result in extra-pulmonary clinical manifestations including encephalopathy or encephalitis [15], [28]. This extrapulmonary dissemination of HPAI H5N1 virus contrasts with seasonal influenza virus infection of humans which, even in fatal cases, is restricted to the respiratory tract. However, there have been relatively few reports describing histopathology and virus distribution in H5N1 cases [5], [16], [21], [24], [26], [27]. To better understand the pathogenesis of human H5N1 virus infection, and investigate the route of virus dissemination in vivo, we report on the use of different techniques to detect virus distribution and infection of 5 organ systems in a laboratory confirmed fatal human H5N1 virus infection, and analyze the relationship between viral load in tissues and host response. Our results suggested that the virus can infect multi-organs besides pulmonary. High viral load is associated with increased host response though the viral load is significantly difference in various organs. Cells of immunologic system could not be excluded to play a role in dissemination of the virus.

## Results

### Virus distribution

Virus culture, real-time RT-PCR, IHC and ISH were used to identify virus distribution in different tissues. As shown in Table 1, live virus was recovered from respiratory tissues including lung, trachea, bronchus and aortopulmonary vessel. In the digestive system, virus was isolated from tissues collected from the ileum, colon and rectum, but not the stomach, duodenum or liver. Of note, virus culture was also positive on tissues collected from brains, ureter and axillary lymph-node. Sequencing results showed that the sequences of isolates are identical. Real time RT-PCR results were consistent with the detection of virus by culture, except for the liver and spleen tissues which were positive by real time RT-PCR but negative for virus isolation. The tissue distribution of viral RNA or antigen detected by ISH and IHC stains respectively, was also generally consistent with virus isolation by culture or real time RT-PCR result. However, several exceptions were noted. There was a lack of detectable staining by either method in bronchus tissue, despite virus detection. On the other hand, both staining methods detected viral product in the kidney, although virus isolation and real time PCR were both negative.

**Table 1:** The results of virus isolation, RT-PCR, ISH and IHC stains in selected tissues.

Sample ID	Organ	Virus isolation	RRT- PCR	ISH		IHC (NP)
				NP	H5	
Respiratory system						
1	Bronchus	+	+	-/-*	-/-	-
2	Trachea	+	+	+/+	+/+	+
3	Aortopulmonary	+	+	+/+	+/+	+
4	Left-up lung	+	+	+/+	+/+	+
5	Left-down lung	+	+	+/+	+/+	+
6	Right-up lung	+	+	+/+	+/+	+
7	Right-middle lung	+	+	+/+	+/+	+
8	Right-down lung	+	+	+/+	+/+	+
Digestive system						
9	Stomach	-	-	-/-	-/-	-
10	Duodenum	-	-	-/-	-/-	-
11	Ileum	+	+	+/+	+/+	+
12	Colon	+	+	+/+	+/+	+
13	Rectum	+	+	+/+	+/+	+
14	Liver	-	+	+/+	+/+	+
Nervous system						
15	Cerebrum cortex	+	+	+/+	+/+	+
16	cerebral medullary substance	+	+	+/+	+/+	+
17	Cerebellum	+	+	+/+	+/+	+
18	Brain stem	+	+	+/+	+/+	+
19	Hippocampus	+	+	+/+	+/+	+
Excretory system						
20	Kidney cortex	-	-	+/+	+/+	+
21	Kidney medulla	-	-	+/+	+/+	+
22	Ureter	+	+	+/+	+/+	+
Lymphoid system						
23	Spleen	-	+	+/+	+/+	+
24	Lymph node	+	+	+/+	+/+	+

*Results presented for sense/antisense probes (identical results for nucleoprotein and haemagglutinin gene). Plus sign  =  positive. Minus sign  =  negative.

### Viral load and host response

**Figure 1:**
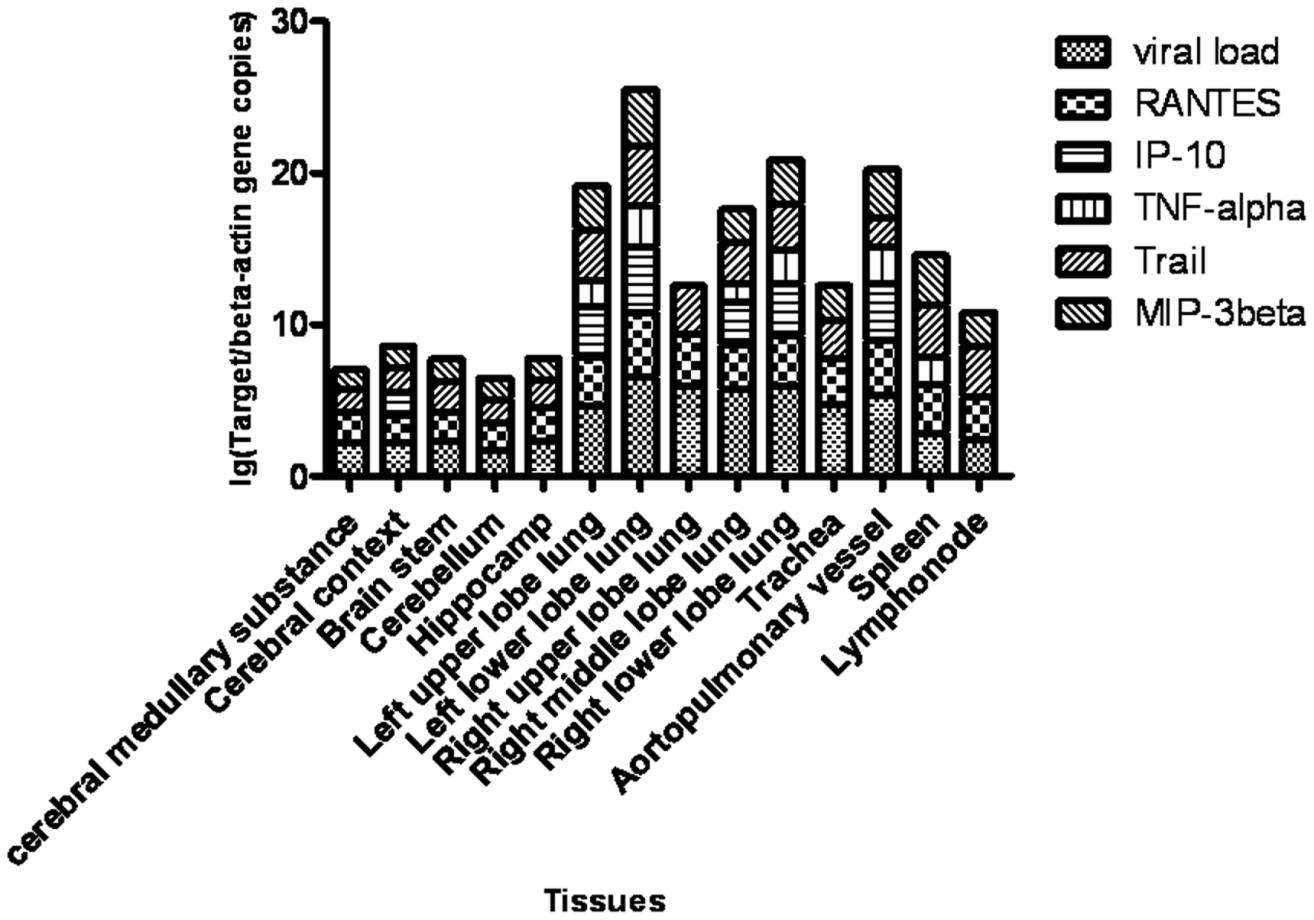
The correlation between viral load and levels of proinflammatory factors in various tissues. Similar with viral load assay, RANTES, IP-10, TNF-α, TRAIL and MIP-3β genes in respiratory tissues, brains, lymph-node and spleen were quantified by real-time RT-PCR. The ratios between proinflammatory factors and β-actin gene copies were presented by logarithm.

The viral load is associated with host response figured out by proinflammatory factors. The relative H5N1 viral load in different tissues by determining the ratio of viral HA copy number relative to the copy number of the beta actin gene for a given tissue sample. Tissues of the respiratory system yielded higher copy number ratios than tissues from all to other organ systems with the lower left lung lobe yielding the highest viral load, overall. Interestingly, ureter tissue had the highest viral load of non respiratory system tissues. All other tissues had lower viral loads that were not. Among the digestive system tissues, the viral load in the liver was the highest (See Fig. S1). Additionally, we detected mRNA copies of macrophage attractant chemokine CXCL10 (IP-10), macrophage inflammatory protein 3β (MIP-3β), RANTES, tumor necrosis factor (TNF)–related apoptosis-inducing ligand (TRAIL) and TNF-α in tissues of brain, respiratory organ, spleen and lymph-node. RANTES and TRAIL can be detected in all selected tissues. MIP-3β is positive in selected tissues except right upper lobe lung with positive β-actin. IP-10 can be determined copies in cerebral context, left upper lobe lung, left lower lobe lung, right middle lobe lung, right lower lobe lung, and aortopulmonary vessel. TNF-α can be showed copies in left upper lobe lung, left lower lobe lung, right middle lobe lung, right lower lobe lung, aortopulmonary vessel and spleen. The correlation among levels of viral load and were showed by Fig. 1. The Pearson's cross correlation analysis (See Table S1) showed that high viral load is associated with high host response figured out by proinflammatory factors (p<0.05) except TNF-α (P>0.05), and the correlation is significant between proinflammatory factors except between TRAIL and TNF-α (p>0.05), and between TRAIL and IP-10(p>0.05) as well.

### Histopathologic features

**Figure 2:**
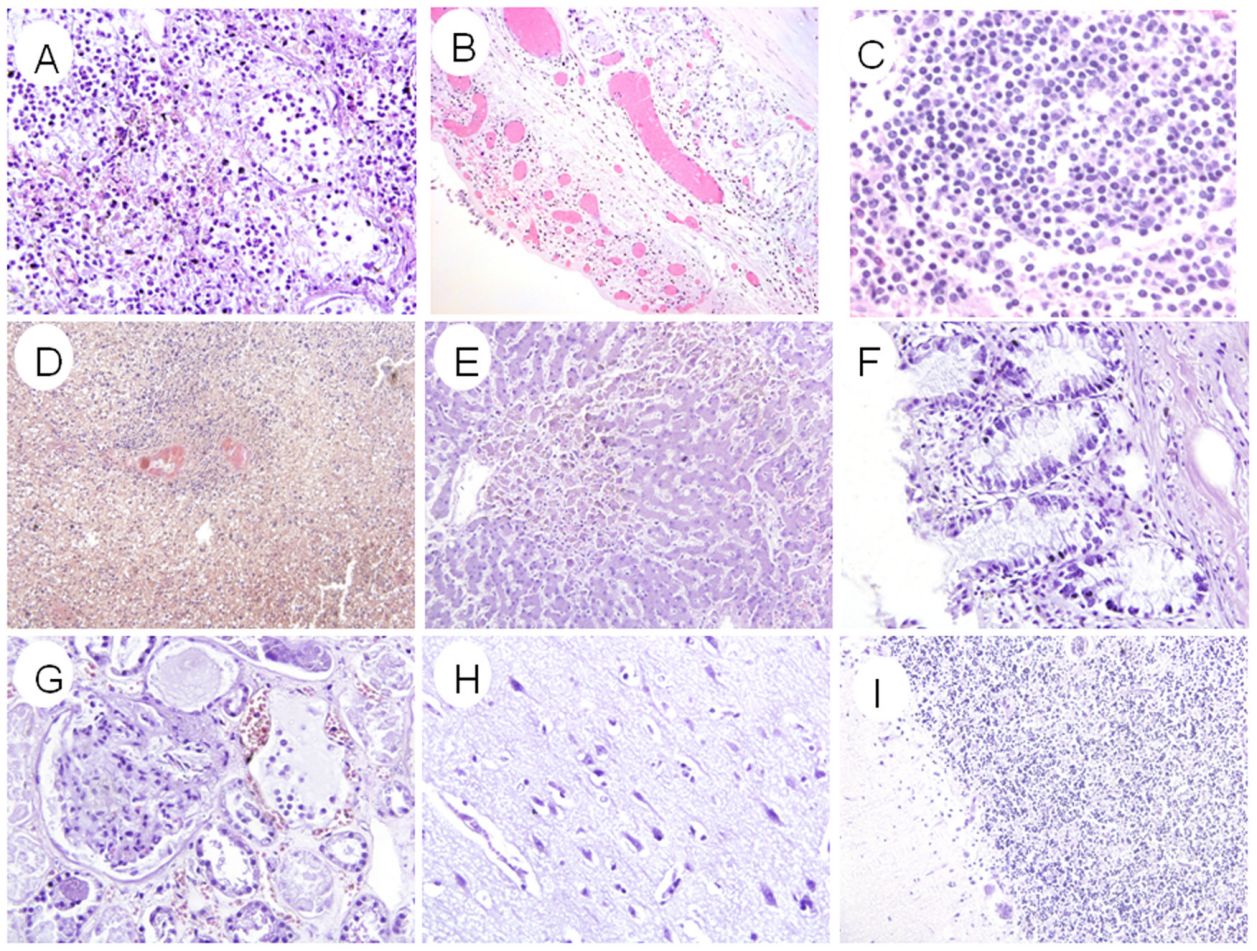
The histopathologic features by H&E stain. (A) Lung tissue showed diffuse alveolar damage characterized by intraalveolar edema, mononuclear inflammation cell infiltration, necrosis of alveolar lining cells, desquamation of pneumocytes in alveolar spaces and hyaline membrane formation. (B) Trachea showed focal denudation of the epithelium with edema and mononuclear inflammatory cell infiltrates. (C) Lymph node was congested with depletion of lymphocytes. (D) Spleen showed depletion of lymphocytes with congestion and organized infarcts. (E) Liver was congested with edema and focal fatty degeneration. (F) Intestine showed mild inflammatory infiltrates in lamina propria. (G) Kidney was congested with edema. (H) Cerebral cortex showed edema with focal neuronal necrosis in hippocampus area. (I) Cerebellum showed focal dilatation between Purkinje cells layer and particle cells layer. Original magnifications: (A–C)×20, (D) ×10, (E–I) ×20.

The histopathologic features in different organs are shown in Fig. 2. Lung showed diffused alveolar damages including intra-alveolar edema, focal intra-alveolar hemorrhage, necrosis of alveolar line cells, focal desquamation of pneumocytes in alveolar spaces, interstitial mononuclear inflammatory cell infiltrates, and extensive hyaline membranes. Trachea showed focal denudation of the epithelium with edema, and mononuclear inflammatory cell infiltrates. Spleen showed depletion of lymphocytes with congestion and organized infarcts. Axillary lymph-node was congested with depletion of lymphocytes. The central nervous system showed extensive edema with focal neuronal necrosis in hippocampus. Diastem between Purkinje cells layer and particle cells layer showed focal augmentation in cerebellum. Liver was congested with edema and focal fatty degeneration. Kidney was congested with edema. Other selected tissues showed no significant histological changes.

### Viral location and cell targets

**Figure 3:**
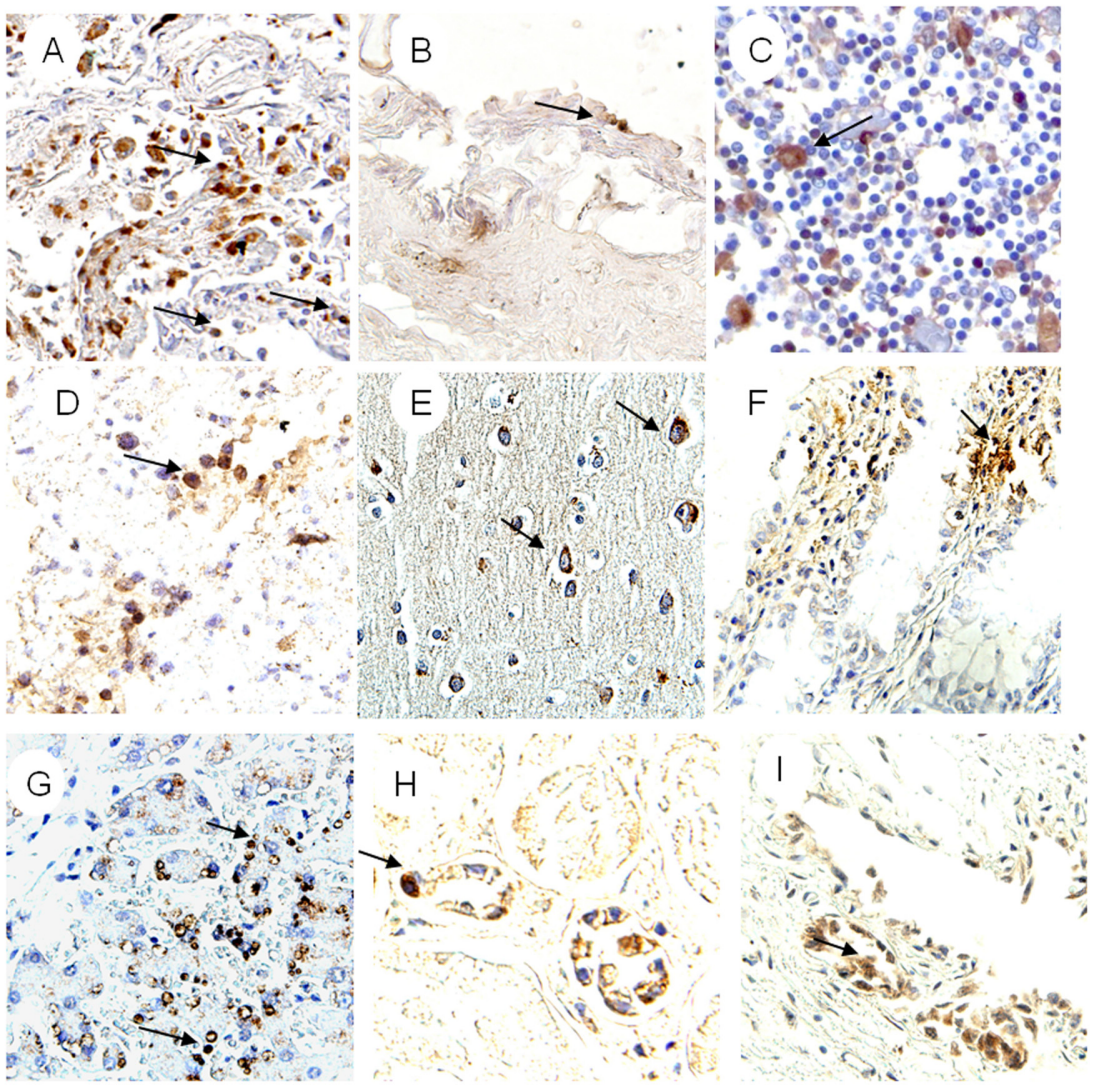
The results of IHC stain with NP against antibody. Positive stain seen with diaminobenzidine (brown; Dako). Slides counterstained with haematoxylin. (A) Positive stain presented in pneumocytes of lung (arrow). (B) Endotheliocyte of aortopulmonary vessel (arrow). (C) Positive stain in nuclei and cytoplasm of axillary lymphoid-node (arrow). (D) Positive stain in lymphocytes of spleen (arrow). (E) Positive stain in cytoplasm of neuron and gliocyte from cerebral cortex (arrows). (F) Positive stain in nuclei and cytoplasm of intestine mucosa (arrows). (G) Positive stain in nuclei of mononuclear-like cells (arrows) with morphological features of macrophages from liver. (H) Positive stain in cytoplasm and nuclei of endotheliocytes of renal contex. (I) Positive stain in nuclei and cytoplasm of epithelial cells from ureter (arrow). Original magnifications: (A) ×20, (B–E) ×40, (F–G) ×20, (H–I) ×40.

**Figure 4:**
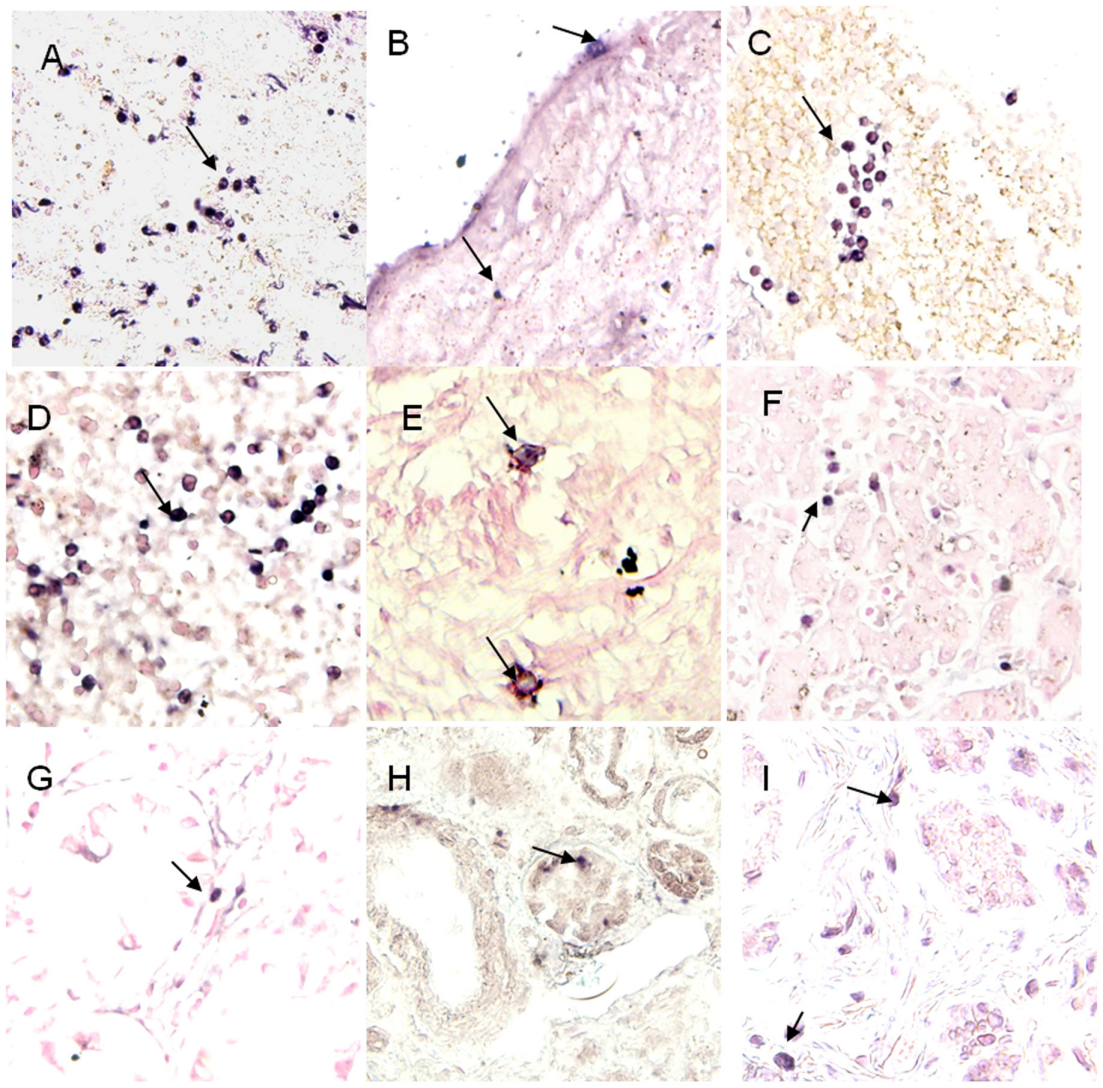
The results of ISH stain with probes of NP gene and HA gene. Positive stain seen with nitroblue tetrazolium/5-bromo-4-choloro-3-indolyl phosphate (purple-blue, Roch). Slides counterstained with nucleic fast red solution. (A) Positive stain (with NP sense probe) in nuclear and cytoplasm of pneumocytes (arrows). (B) Positive stain (with NP antisense probe) in nuclei and cytoplasm of Endotheliocyte from aortopulmonary (arrows). (C) Positive stain (with NP antisense probe) in lymphocytes of oxter lymph-node (arrows). (D) Positive stain (with NP antisense probe) in lymphocytes and monocytes from spleen (arrows). (E) Positive stain (with NP antisense probe) in nuclei and cytoplasm of neuron from Cerebrum cortex (arrow). (F) Positive stain (with NP antisense probe) in cytoplasm of epithelial cells from Colon (arrow). (G) Positive stain (with NP antisense probe) in lymphocytes of liver (arrow). (H) Positive stain (with HA antisense probe) in cytoplasm of endotheliocyte of renal contex (arrow). (I) Positive stain (with NP antisense probe) in nuclei of epithelial cells of ureter. Original magnifications: (A) ×20, (B–I) ×40.

**Figure 5:**
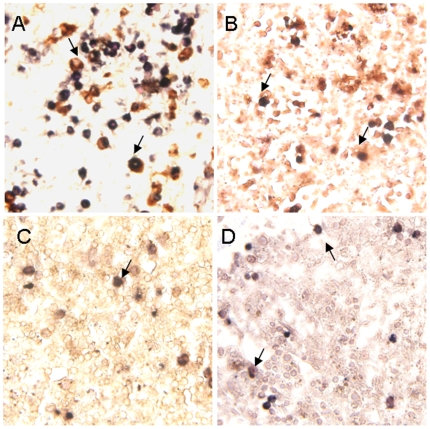
Double-labeled stain combined ISH for HA gene and IHC for the cell superfic markers of macrophage CD68 protein, T lymphocyte CD3 protein, progenitor cells CD34 protein and follicular dendritic cells CD35 protein. Positive of in-situ hybridization seen with nitroblue tetrazolium/5-bromo-4-choloro-3-indolyl phosphate (purple-blue, Roch), IHC with diaminobenzidine (brown; Dako). Slides counterstained with 2% nuclear fast red solution. (A) Double positive cells in lung. The viral sequence presented in nucleic and cytoplasm of CD68^+^ cell (arrow). Viral sequence presented in nucleic and cytoplasm what showed the feature of endocytosis. (B) Viral sequence positive stain presented in CD3^+^ T lymphocytes of spleen (arrow). (C) Viral sequence positive signal presented in CD34^+^ cell of spleen (arrow). (D) Viral sequence positive stain presented in CD35^+^ cell of spleen (arrow). Original magnifications: (A–D) ×40.

Viral location in tissues was determined by IHC and ISH stains. IHC stain showed, In addition to pneumocytes and epithelial cell of trachea, positive immunostaining with nucleoprotein antibody was found in endotheliocyte of aortopulmonary vessel (Fig. 3B), lymphocytes of lymph-node (Fig. 3C) and spleen (Fig. 3D), neuron of brain (Fig. 3E), intestinal cells(Fig. 3F), epithelial cells in liver(Fig. 3G), kidney(Fig. 3H) and ureter cells (Fig. 3I). As influenza virus is a negative-strand RNA virus, ISH stain with sense and antisense probes detected both virus RNA (with sense probe), mRNA and cRNA(with anti-sense probe). ISH stain showed that the two sets of probes (for hemagglutinin and nucleoprotein) generated similar staining results. In the selected 24 autopsy tissue from 5 organ systems, positive staining of ISH was detected in all samples except Bronchus, stomach and duodenum. In the lung tissue samples, sense probes extensively hybridized in the nuclei of pneumocytes (Fig. 4A), whereas antisense probes hybridized in both the cytoplasm and nuclei. In all other organs with positive staining, both sense and antisense were present mainly in the cytoplasm of infected cells. In respiratory system, the positive staining was found in epithelial cell of lung (Fig. 4A), trachea and aortopulmonary vessel (fig. 4B), but was not found in bronchus. The positive staining also presented in lymphocytes of lymph-nodes (Fig. 4C), and spleen (Fig. 4D), neuron of brains (Fig. 4E), epithelial cell of intestines (Fig. 4F), epithelial cell of liver (Fig. 4G), and kidney (Fig. 4H), mononuclear cells and epithelial cell of ureter (Fig. 4I). Additionally, Double staining combined with ISH and IHC revealed that immune cells in selected tissues involved with H5N1 virus RNA were macrophages (co-labeled with CD68 fig. 5A), T lymphocytes (co-labeled with CD3 fig. 5B), progenitor cells (co-labeled with CD34 fig. 5C) and follicular dendritic cells (co-labeled with CD35 fig. 5D).

## Discussion

We systematically studied the tissue tropism of H5N1 virus in a fatal human case, based on 24 autopsy tissue samples from 5 organ systems, and analyzed the relationship between viral load and host response level in selected tissues. We presented evidence suggesting that the H5N1 virus can infect selected tissue of selected 5 organ systems including respiratory, digestive, nervous, urinary and lymphoid system. High viral load can induce high proinflamatory factors level in tissues, and immune cells could be the target of the virus.

There is a substantial amount of evidence that HPAI H5N1 virus can infect extrapulmonary organ tissues [16]–[18], [27] and precede other clinical manifestation [20], [29]. Our results presented that the viral antigens or viral RNA can be found in trachea, lung, brain, intestines, liver, spleen, lymph-node and kidney which were reported as same before [20], [26], [27], [30], as well as in aortopulmonary vessel and ureter which were not reported before. Notably, the virus can be found in tissues of lower gastrointestinal tract including small intestine and large intestine but in stomach and duodenum. The origin of infection in the extrapulmonary organs could be blood-borne, which is supported by previous studies showing live H5N1 virus can be isolated from the serum [20] and plasma [31]. Unfortunately, we haven't obtained blood samples to perform viral detection. However, other routes for virus dissemination in vivo, such as circulating immune cell that has been postulated to be involved in the pathogenesis of SARS [32], can not be excluded because previous in-vitro and/or in-vivo results have showed that the virus can replicate in multiple immune cells including monocyte and macrophage [25], [30], [33]–[36] and T lymphocytes [37].

The viral load shows various levels in different organs although the virus can be found in multiple organs. Quantitative detection of viral gene showed that viral load is the highest in tissues of respiratory system, especially, in left lower lobe of lung. The liver had the highest viral load in tissues of digestive system. Interestingly, renal duct showed a high viral load although PCR detection of kidney was negative. And viral load was high in spleen and lymphonode. Despite of the viral cells tropism, possible reasons of different viral load could include activation of low PH [38], viral receptor distribution [39], cell N-linked glycoprotein distribution [40] or other unknown mechanisms. On other hand, non-permissive or abortive infection is extra possible in some of the tissues where the virus cannot replicate effectively. Additionally, it should be possible that phagocytosis of viral antigen from pathologically significant lytic/replicative infection caused positively viral detection in these tissues.

High viral load is associated with increased host responses. To be different with seasonal influenza virus, the H5N1 virus caused intense transcription induction and secretion of inflammatory cytokines/chemokines, as documented in human cases [15], [22], and increased induction of cytokines and chemokines has been demonstrated in H5N1-infected mice [41]. Moreover, High virulence of H5N1 virus is associated with increase with increased host responses [42]. However, the relationship between viral load and host immuno-response has never been reported. Our study suggested that the high viral load is correlated with proinflamatory factors including IP-10, RANTES, MIP-3β and TRAIL. This finding is particularly relevant to the mechanism of H5N1 pathogenesis which is associated not only with the virus but also with the host responses. Inflammatory protein can be produced by almost any infected cells and by immune cells, including alveolar macrophages [43]. However, type II pneumocytes have a marked ability to secrete large amounts of cytokines, such as TNF-α, GM-CSF, MCP, and IL-8, in response to various insults [44], [45] and can be induced to secrete IL-1α, IL-6, RANTES, and MCP-1, in response to TNF-α [45], [46], the last being produced by alveolar macrophages during H5N1 infection. Therefore, it is possible that the much greater cytokine induction by the H5N1 virus was as much a consequence of the response of individual, infected cells as it was a consequence of the numbers of infected cells.

Immune cells could be a target of the virus infection. Previous findings showed that viral sequences and antigens have been detected in lymphocytes in lymph node tissue, as well as in Hofbauer cells (macrophages of the placenta), Kupffer cells (macrophages of the liver), and mononuclear cells in the intestinal mucosa [27]. This is consistent with our find. Our presented evidence shows that virus can be detected in macrophage. Moreover, our finding showed that viral RNA can be detected in CD3+ T lymphocytes, progenitor cells and follicular dendritic cells of spleen. The spleen is a complex organ with several functions, including the removal of senescent or aberrant red blood cells from circulation, as well as the removal of circulating pathogenic organisms [47]. So circulationg immune cell with H5N1 virus could play a role in dissemination of virus when those cells with virus can not remove the virus in cells completely.

We unprecedentedly investigated 24 autopsy tissues of 5 organ systems from a dead patient of H5N1 infection. Our study results indicate that the H5N1 virus could be found in multiple organs. Viral load in infected tissues is correlated with host response. And circulating immune cell can not be excluded to play a role in dissemination of virus in vivo.

## Materials and Methods

### Patient

The patient was laboratory confirmed as an H5N1 infection by China CDC on February 20, 2008. He is a 42 years-old Chinese male living in Guangxi, China with a history of 6 days of fever, cough and dyspnea. Two weeks before hospital admission, he bought 3 hens at an open-air market, one of which died later at same day. The remaining birds exhibited symptom of chicken attack next day. The patient killed and cooked the birds, and ate them together with other family members who later did not experience any clinical symptom. On admission, the patient was febrile with a temperature of 40.3°C, had infiltration of lower left lung lobe based on chest radiography, bilateral lower lung moist rales, and substantially reduced oxygen saturation. He was placed on a ventilator and treated with antibiotics, spasmolysis, corticosteroids, and the dissipatation of phlegm and fluids. Despite treatment, the patient presented with function damage of multiple organ (lung, heart, liver and kidney) on the second day after admission, and died 59 h after admission, and 8 days after the onset of symptoms. Throat swabs were collected and performed RT-PCR and rRT-PCR detection at the day patient died. No antiviral drug treatment was given since the patient died before finally laboratory diagnosis.

After informed consent was obtained, the cadaver was stored at 4°C and underwent autopsy about 18 h after death. The autopsies were done following conventional protocols with strict adherence to biosafety procedures [48]. Twenty-four tissues were collected from respiratory, digestive, nervous, urinary and lymphatic organ systems. Duplicate tissue samples were collected; one sample was fixed in diethylpyrocarbonate (DEPC) treated 10% formalin for pathologic analyses, while a second sample was frozen at −80°C for virus isolation and molecular analyses.

### Virus isolation

Frozen tissues were thawed and homogenized before inoculation into embryonated eggs for viral culture. Briefly, 1.0 ml of phosphate-buffered saline (PBS) was added to 100–150 mg of tissue, which was homogenized, and, then centrifuged; 0.1 ml of the recovered supernatant was injected into the allantoic cavity of 11 days-old specific pathogen-free embryonated chicken eggs. The allantoic fluids were tested for haemagglutation activity with turkey red blood cells after 72 h incubation at 35°C. Samples containing virus were subjected to RT-PCR and sequencing. Three blind passages were performed on hemagglutination-negative samples to confirm the absence of infectious virus. All steps were performed in BSL-3 containment laboratory.

### RNA extraction

Total RNA from the tissues samples was extracted using an Axygen total RNA extraction kit (Axygen, USA) as described in the manufacturer's instructions. Briefly, 400 µl of lysis buffer was added to 30–40 mg of ground tissue. The nucleic acids were eluted in 50 µl nuclease-free water and stored at −20°C. For every five samples, one negative control (water) was included to detect any possible contamination.

### Standard RNA synthesis

Control RNA products derive from in vitro transcription of the matrix (M) gene RNA of A/Anhui/1/2005(H5N1), human house keeping gene beta–actin and human cytokines/chemokines (including IP-10, RANTES, TRAIL, MIP-3β and TNF-α) genes were used as positive controls and to establish the detection limit of the assay. Recombinant plasmids with entire M gene, beta–actin gene segment and cytokines/chemokines gene segment were linearised by restriction enzyme, and then purified using a DNA clean-up kit. DNA concentration was measured as OD units at 260 nm. One µg of linearized plasmid DNA was transcribed using Riboprobe in vitro transcription system kit (Promega, USA) according to the manufacturer's instructions. The transcribed RNA was purified using phenyl/chloroform solution and was quantified by spectrophotometer. RNA copy number was then determined following the method of Fronhoffs [49].

### Viral load and proinflammatory factors quantitative assay

To analyze the H5N1 viral load and quantify proinflammatory factors in different tissues, real-time RT-PCR was performed with a Strategene detection system using a fluorescently labeled TaqMan probe to enable continuous monitoring of amplicon formation. The primer and probe of H5 HA gene is from the WHO-released primer sets [50]. The primers and probes of proinflammatory factors and beta-actin gene were obtained from the literature [51], [52]. The concentration of primer and probe used was 40 µM and 10 µM, respectively. The reaction was completed in a total volume of 25 µl performed by QuantiTect Probe PCR Kit (Qiagen, Germany). The reaction mixture was incubated with 5 µl DNase-treated total RNA (the template which was used to amplify β-actin gene was performed by 100-fold dilution) at the following temperature cycles. First, the reverse transcription reaction was completed by 1 cycle at 50°Cfor 30 min. Next, H5N1 HA gene, cytokine/chemokine gene and house keeping (β-actin) genes were amplified by 1 cycle at 94°C for 15 min and 45 cycles at 94°C for 15 s, 55°C for 30 s, and 72°C for 30 s each. As described in previous report [53], the standard curve was generated using serial dilution of in vitro transcribed standard RNA (from ∼10 to 10^7^ copies). The viral load and cytokines/chemokine levels are presented as the log_10_ value of the ratio between copies of the target gene and β-actin gene.

### Immunohistochemistry (IHC)

Immunohistochemical stain was performed on 5 µm thick deparaffinized sections using monoclonal antibodies against the nucleoprotein of influenza A (Serotec, UK) by a two-step peroxidase method. For controls, we used unrelated antibodies in place of the primary antibody. Briefly, sections were deparafinized by 2 washes in xylene and were rehydrated through decreasing concentrations of ethanol. After washing in PBS at pH 7.6 for 5 min at room temperature (RT), sections were heat-treated with antigen-retrieval solution (TRIS/EDTA buffer, pH 9, Dako, Denmark) for 10 min using microwave antigen retrieval method. After blocking with 10% normal horse serum for 10 min at RT, the sections were incubated with the specific antibody (1∶100) for 30 min at RT. Unbound antibody was removed by 3 washes in PBS before adding HRP-labelled polymer for 30 min at RT (Dako CSA Detection System, Denmark). After washing unbound labeled polymer in PBS 3 times, peroxidase staining in tissue sections was revealed by DAB solution (CSA Detection Systems, DAKO, Denmark). After stopping the reaction in running water, sections were counter-stained with a quick rinse in Mayer's hematoxylin solution. After dehydrating with increasing concentrations of ethanol and xylene, the sections were mounted with DPX and examined by light microscopy (Olympus BX51, Japan).

### In-situ hybridization (ISH)

The development of probes was based in analysis of the full haemagglutinin and nucleoprotein gene sequences of all Chinese human isolates of H5N1. Oligonucleotide DNA probes representing conserved gene regions were used. Probes were labeled by digoxigenin-UTP (Roche Diagnostics, Penzberg, Germany) and tested for specificty using human biopsy of gut tissue with in vitro infection of H5N1 virus. Since H5N1 is a negative-stranded RNA virus, sense probes were defined as the probes that detect the viral RNA (negative-stranded), whereas antisense probes detected mRNA and complementary RNA (cRNA), which are both positive-stranded. Briefly, before hybridization, all solutions were prepared with DEPC-treated water. After deparaffinization and rehydration, tissue sections of 5 µm thickness were treated with proteinase K digestion for 30 min. Tissue sections were then incubated with a hybridization cocktail containing 25 µg/mL of probes at 37°C for 16∼18 h. All sense and antisense probes were applied separately on consecutive tissue sections. After blocking with normal horse serum (1∶100), sections were incubated with alkaline phosphatase-labelled digoxigenin antibody (1∶1000, Roche Diagnostics, Penzberg, Germany) for 1 h, and the reaction products were colorised with nitroblue tetrazolium/5-bromo-4-choloro-3-indolyl phosphate (Roch Diagnostics, Penzberg, Germany) for 1–1.5 h, and counterstained in 2% nucleic fast red liquid for 1 min. As a positive control, we used human lung and gut biopsy tissues with in-vitro infection of H5N1 virus. Negative controls also included an unrelated antisense probe against the fragment of the heamaglutinin gene of the seasonal influenza virus H3N2 as well as H5N1 in-situ hybridization probes to tissues.

### Double labeling with ISH and IHC

After completeing the colorization reaction of the ISH as outlined above, sections were incubated with 3% hydrogen peroxide to quench endogenous peroxidase activity. The sections were then blocked with 10% normal horse serum for 10 min at RT, before incubating with monoclonal antibodies (against CD3^+^, CD68^+^, CD34^+^, D35^+^) for 1 h at RT. Unbound antibody was removed by 3 washes in PBS before the addition of HRP-labeled polymer for 30 min at RT (Dako CSA Detection System, Denmark). After washing 3 times with PBS to remove unbound labeled polymer, peroxidase staining in tissue sections was revealed by DAB solution (CSA Detection Systems, DAKO, Denmark). After stopping the reaction in running water, sections were counter-stained by 2% nuclear quick red solution for 30 s. For each tissue, double-labeled stain with IHC and ISH was repeated three times for data analysis. To strengthen further the results of colocalization studies, we performed ISH and IHC on consecutive sections. Tissue sections showing ISH-positive cells were carefully compared with consecutive tissue sections on which IHC with antibodies against specific cell markers was applied. Co-localization of a specific cellular marker and viral genome was clearly identified.

### Statistical analysis

The correlation between viral load and quantitative proinflammatory factors profile was analyzed by Pearson's correlation test using Instat software (Vision 5.0, GraphPad prism). Differences were considered significant at p<0.05.

## Supporting Information

Figure S1 The distribution of viral load in selected tissue samples. The viral HA gene and β-actin gene copies in tissues were determined by quantified real-time RT-PCR. The ratios between HA and β-actin gene copies which was showed by logarithm presented the viral-load level in different tissue.
(0.11 MB TIF)

Table S1 The Pearson's correlation coefficient among viral load in tissues and host response.
(0.04 MB DOC)
