# Supplementary figures and images for: A Systematic Molecular Pathology Study of a Laboratory Confirmed H5N1 Human Case
Source: PLoS One. 2010 Oct 12;5(10):e13315. doi: 10.1371/journal.pone.0013315 (PMC2953511; doi:10.1371/journal.pone.0013315)

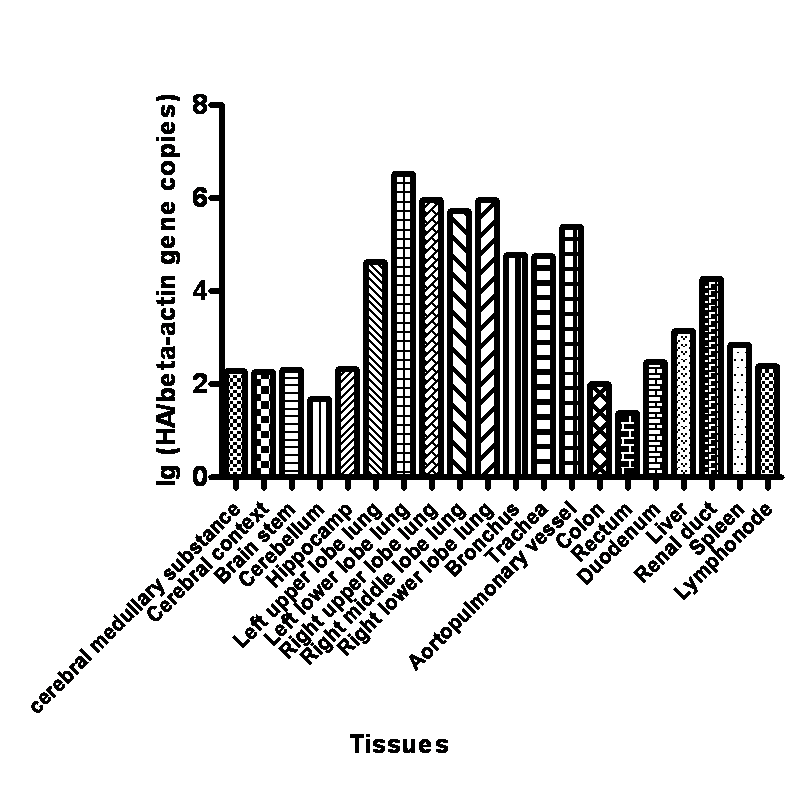

Supplement: Figure S1 — The distribution of viral load in selected tissue samples. The viral HA gene and β-actin gene copies in tissues were determined by quantified real-time RT-PCR. The ratios between HA and β-actin gene copies which was showed by logarithm presented the viral-load level in different tissue. (0.11 MB TIF) [file pone.0013315.s001.tif]
