# Supplementary material for: A Systematic Molecular Pathology Study of a Laboratory Confirmed H5N1 Human Case
Source: PLoS One. 2010 Oct 12;5(10):e13315. doi: 10.1371/journal.pone.0013315 (PMC2953511; doi:10.1371/journal.pone.0013315)
Supplement: Table S1 — The Pearson's correlation coefficient among viral load in tissues and host response. (0.04 MB DOC) [file pone.0013315.s002.doc]

Table. S1 The Pearson’s correlation coefficient among viral load in tissues and host response

|  | Viral load | | RANTES | | IP-10 | | TNF-α | | Trail | | MIP-3β | |
| --- | --- | --- | --- | --- | --- | --- | --- | --- | --- | --- | --- | --- |
|  | CrC* | P^†^ | CrC | P | CrC | P | CrC | P | CrC | P | CrC | P |
| Viral load |  |  | 0.857 | 9E-05 | 0.89 | 0.017 | 0.38 | 0.457 | 0.585 | 0.028 | 0.761 | 0.003 |
| RANTES | 0.857 | 9E-05 |  |  | 0.992 | 9E-05 | 0.889 | 0.018 | 0.813 | 4E-04 | 0.961 | 2E-07 |
| IP-10 | 0.89 | 0.017 | 0.992 | 9E-05 |  |  | 0.96 | 0.01 | 0.703 | 0.119 | 0.991 | 1E-04 |
| TNF-α | 0.38 | 0.457 | 0.889 | 0.018 | 0.96 | 0.01 |  |  | 0.217 | 0.679 | 0.877 | 0.022 |
| Trail | 0.585 | 0.028 | 0.813 | 4E-04 | 0.703 | 0.119 | 0.217 | 0.679 |  |  | 0.817 | 7E-04 |
| MIP-3β | 0.761 | 0.003 | 0.961 | 2E-07 | 0.991 | 1E-04 | 0.877 | 0.022 | 0.817 | 7E-04 |  |  |

Note: *CrC is the abbreviation of correlation coefficient. ^†^P represents p value of pearson’s correlation coefficient.
